# Editorial: Multiple concussions: from preclinic to clinic

**DOI:** 10.3389/fneur.2026.2001679

**Published:** 2026-10-29

**Authors:** Giuseppe Lazzarino, Stefano Signoretti, Valentina Di Pietro

**Affiliations:** 1 LTA-Biotech srl, Paternò (CT), Italy; 2 Departmental Faculty of Medicine, UniCamillus, Saint Camillus International University of Health and Medical Sciences, Rome, Italy; 3 Sant'Eugenio Hospital, Rome, Italy; 4 Neurotrauma and Ophthalmology Research Group, Institute of Inflammation and Aging, University of Birmingham, Birmingham, United Kingdom

**Keywords:** brain dysfunctions, single or multiple concussion, metabolic brain vulnerability, molecular biomarkers, return to play, neuroinflammation, chronic traumatic encephalopathy, sports-related concussion

Concussion is increasingly recognized not simply as a transient clinical event, but as a complex biological process in which the apparent resolution of symptoms may not necessarily coincide with complete recovery of the brain. This distinction becomes particularly important when concussion occurs repeatedly, or when individuals are exposed to multiple sub-concussive head impacts that may not result in an overt clinical diagnosis. Multiple Concussions: From Preclinic to Clinic, builds on the growing body of evidence in this rapidly evolving field and brings together Pre-clinical, clinical and translational perspectives to address one of the most challenging questions in concussion research: when does repeated exposure cease to be transient and begin to produce persistent brain dysfunction?

A key challenge in concussion management remains the absence of a readily accessible measure of biological recovery. Clinical assessment continues to rely heavily on symptoms, neurological examination and neurocognitive function, yet these measures may normalize while molecular, metabolic and cellular alterations persist. This creates a potential mismatch between the clinical recovery of the individual and the biological recovery of the brain. Understanding this distinction is particularly relevant when decisions regarding return-to-play, return-to-learn or return to other activities are made in the context of continued exposure to head impacts (Bevilacqua et al.).

The contributions to this Research Topic address this problem from complementary perspectives. A first important theme is the search for objective biomarkers of concussion and recovery. Molecular biomarkers have the potential to provide information that cannot be obtained from symptoms alone, particularly if they can distinguish acute injury from persistent or cumulative effects. The studies included in this collection contribute to the growing effort to identify molecular signatures associated with repeated concussion and to determine whether these signatures correlate with clinical symptoms, cognitive performance and recovery trajectories (Le Belle et al.). Importantly, future biomarkers will need to demonstrate not only diagnostic value, but also the ability to inform prognosis and, ultimately, individualized clinical decision-making (Napora et al.).

A second major theme concerns sub-concussive exposure. The absence of clinically recognized concussion does not necessarily imply the absence of biological consequences. Athletes in contact and collision sports may experience numerous head impacts over a season or across an entire sporting career without reporting symptoms. Determining whether the cumulative burden of these exposures produces measurable and clinically meaningful alterations remains a critical research priority. Contributions examining sub-concussive exposure therefore challenge the traditional binary distinction between “concussion” and “no concussion” and highlight the need to consider head-impact exposure as a continuum.

Pre-clinical models provide another essential component of this continuum. Repeated-injury paradigms allow researchers to investigate questions that are difficult to address clinically, including the relationship between number, interval and severity of impacts and subsequent pathological outcomes. An important unanswered question is whether there is a threshold—or perhaps a more complex interaction between injury burden, timing and individual susceptibility—beyond which the brain fails to return to its previous state. Rather than searching for a universal number of concussions that is “too many,” future research may need to identify the biological conditions under which repeated injury becomes increasingly harmful.

These models are also essential for understanding the mechanisms linking repetitive head trauma with persistent neuroinflammation, metabolic dysfunction, axonal and synaptic abnormalities, and neurodegenerative processes. In particular, the relationship between repetitive traumatic brain injury and chronic traumatic encephalopathy (CTE) remains an area of considerable scientific interest and debate. Importantly, the presence of repetitive head impacts alone should not be assumed to predict an inevitable neurodegenerative outcome. Understanding why some individuals appear resilient whereas others develop persistent neurological or neuropsychiatric consequences will require integration of injury characteristics with biological, genetic, environmental and lifestyle factors.

The clinical implications of these questions are substantial. Current approaches to return-to-play and return-to-learn increasingly recognize that recovery should be individualized, progressive and multidimensional. Nevertheless, clinicians still lack robust tools capable of determining whether an apparently recovered brain remains biologically vulnerable. Contributions addressing clinical management and educational reintegration therefore provide an important bridge between mechanistic research and the realities faced by athletes, students, families, clinicians and sporting organizations.

Taken together, the articles in this Research Topic reinforce the need to move beyond a simple symptom-based model of concussion. The future of concussion research will require integration rather than isolation (Napora et al.): molecular biomarkers with clinical assessment; experimental models with longitudinal human studies; individual concussions with cumulative exposure; and acute recovery with long-term outcomes.

Perhaps most importantly, “how many are too many?” may ultimately prove to be the wrong question. The more clinically relevant question may be: “How many are too many for this individual, at this particular stage of recovery? (Remigio-Baker et al.)” Answering that question will require sensitive measures of biological vulnerability, better characterization of cumulative exposure and a stronger understanding of the mechanisms that determine recovery or progression.

Multiple Concussions: From Pre-clinic to Clinic, contributes to this ongoing effort by bringing together evidence across the translational spectrum. The ultimate goal is not simply to identify the consequences of repeated head impacts, but to develop the knowledge and tools necessary to recognize vulnerability, guide safer recovery and prevent potentially avoidable long-term consequences.

